# An innovative piston corer for large‐volume sediment samples

**DOI:** 10.1002/lom3.10124

**Published:** 2016-07-13

**Authors:** Ivo Gallmetzer, Alexandra Haselmair, Michael Stachowitsch, Martin Zuschin

**Affiliations:** ^1^ Department of PalaeontologyUniversity of ViennaViennaAustria

## Abstract

Coring is one of several standard procedures to extract sediments and their faunas from open marine, estuarine, and limnic environments. Achieving sufficiently deep penetration, obtaining large sediment volumes in single deployments, and avoiding sediment loss upon retrieval remain problematic. We developed a piston corer with a diameter of 16 cm that enables penetration down to 1.5 m in a broad range of soft bottom types, yields sufficient material for multiple analyses, and prevents sediment loss due to a specially designed hydraulic core catcher. A novel extrusion system enables very precise slicing and preserves the original sediment stratification by keeping the liners upright. The corer has moderate purchase costs and a robust and simple design that allows for a deployment from relatively small vessels as available at most marine science institutions. It can easily be operated by two to three researchers rather than by specially trained technicians. In the northern Adriatic Sea, the corer successfully extracted more than 50 cores from a range of fine mud to coarse sand, at water depths from three to 45 m. The initial evaluation of the cores demonstrated their usefulness for fauna sequences along with heavy metal, nutrient and pollutant analyses. Their length is particularly suited for historical ecological work requiring sedimentary and faunal sequences to reconstruct benthic communities over the last millennia.

The extraction of marine or limnic sediments by means of a coring device is a widely used method in aquatic ecology, geochemistry and environmental science, as well as for related historical and palaeontological studies (Blomqvist 1991). As opposed to dredges, grabs and suction samplers, sediment corers ideally preserve the primary depositional structure of the sediment along with the original depth distribution of the enclosed fauna and faunal remains such as shells or skeletal parts. This yields an undisturbed sedimentary archive for investigating past climatic and environmental changes as well as benthic or planktonic community successions over time. Beyond smaller hand‐held, diver‐operated corers, a wide range of larger corers are available. Depending on the functional mechanisms that ensure penetration into the sediment, they can be grouped into the following categories: gravity‐type penetration corers, percussion corers, vibracorers and drill corers (see e.g., Glew et al. 2001; Tetra Tech EM Inc. 2003; Adachi et al. 2010). According to this distinction, many corer types such as box corers, Kasten‐corers, multicorers, Slo‐corers and piston corers can be considered as sub‐types of a gravity corer, because all are driven into the sediment by means of a heavy driving weight. All corers must overcome the hurdle of sufficient depth penetration and loss of material upon extraction from the substrate. The classical gravity corer, an open‐barrel system with a driving weight and a more or less sophisticated closing mechanism, is the most common type and exists in many different variations (Glew et al. 2001; Renberg and Hansson 2008). Its advantages are a relatively simple design, moderate costs, especially for lighter versions, and the fact that it is theoretically unrestricted by water depth. A limitation of classical gravity corers is that penetration depths typically decrease with increasing grain size and core barrel diameter. This makes them less suitable for studies requiring greater amounts of material or longer, multiple cores of equal length in sediments of different caliber (Nesje 1992; Adachi et al. 2010). Furthermore, gravity corers are prone to disturb the water‐rich uppermost sediment layers upon impact due to the shock wave they generate during descent (Burke 1968; McIntyre 1971; Parker and Sills 1990; Blomqvist 1991; Glew et al. 2001; Chaney and Almagor 2015). They may also produce considerable entry deficits (penetration minus sample entry), which can “shorten” the retrieved sediment column by up to 50% (Wright 1993; Skinner and McCave 2003). Another problem is the retention of the core upon extraction, especially in large‐diameter cores (Nesje 1992; Patmore et al. 2014). In piston corers, the action of the piston reduces internal friction, facilitates sample entry and prevents core shortening and plugging (Blomqvist 1991; Wright 1993; Mudroch and MacKnight 1994). This enables longer, less disturbed and more complete cores than those obtainable from simpler gravity corers. Piston corers equipped with a percussion or hammering system improve penetration in stiffer sediments where weight alone may not be sufficient. Such percussion systems are driven by lifting and dropping a weight either by hand or using a winch, or by the action of pneumatic hammers: they help make the whole coring device simpler, faster and cheaper than vibracorers (Jones et al. 1992; Adachi et al. 2010). The increased core lengths and diameters obtainable with piston corers demand effective core catcher systems that prevent partial or complete post‐extraction losses, especially during the critical phase of retrieval from the water. Such core catchers range from the simple “orange peel” (concentric flexible steel lamellae) to the ball catcher type and to more complex systems. Vibracorers penetrate compact sediments, rely on single or multiple electrical motors that provide the vibration, and are connected to the on‐board control unit by special umbilicals. Although capable of producing cores of considerable length, vibracorers (and drill corers) are not a viable solution for many research projects: Sampling locations may be too remote or inaccessible to convey these bulky samplers, appropriately large vessels may be unavailable, or the costs too high.

The coring device presented here was specifically designed for a project on the historical ecology of the northern Adriatic Sea. In this project, we investigate down‐core changes in the structure of molluscan death assemblages: These serve as a proxy for ecological shifts during the time span covered by a sediment core (Kidwell 2007, 2009; Kowalewski 2009; Kosnik et al. 2015). The project is designed to reconstruct the timing and extent of major environmental shifts in the past, and to back‐trace pre‐anthropogenic benthic communities which may be used as a reference for conservation and management efforts. The cores needed to deliver enough shell material for community studies, but also sediment for analyses including granulometry, concentrations of nutrients, heavy metals and organic pollutants, as well as for radiometric sediment dating using Pb‐210. Our basin‐wide approach involved several sampling stations covering different sediment types, nutrient conditions and degrees of protection from bottom trawling, thus incorporating a wide range of benthic habitats. The coring device had to meet the following requirements:

- reliably deliver 1.5‐m‐long cores from all stations, regardless of sediment composition; sedimentation rate data from the literature suggested that a length of 1.5 m should ensure a time coverage of at least a century in high‐ and several millennia in low‐sedimentation settings (Ogorelec et al. 1991; Cattaneo and Trincardi 1999);
- extract cores with a diameter of 16 cm (a common measure for standard translucent PVC tubes that serve as liners) to provide enough material for shell community analyses;
- efficiently retain a variety of sediment types from soft muds to sands despite the large core diameter;
- enable quick and easy deployment to yield multiple cores in a timely manner;
- be used in combination with a core‐extruding system that guarantees a permanent upright position of the heavy cores during liner extraction and preparation for slicing, as well as a precise slicing of the cores directly on board;
- be relatively affordable and allow handling by the researchers themselves, potentially using a smaller, modestly equipped boat as available at most research stations.

The UWITEC Company based in Mondsee, Austria, conceived and designed the coring device meeting the above specifications. After several test drives and minor adjustments, we applied it successfully in the field.

## Materials and procedures

### Working principle

**Figure 1:**
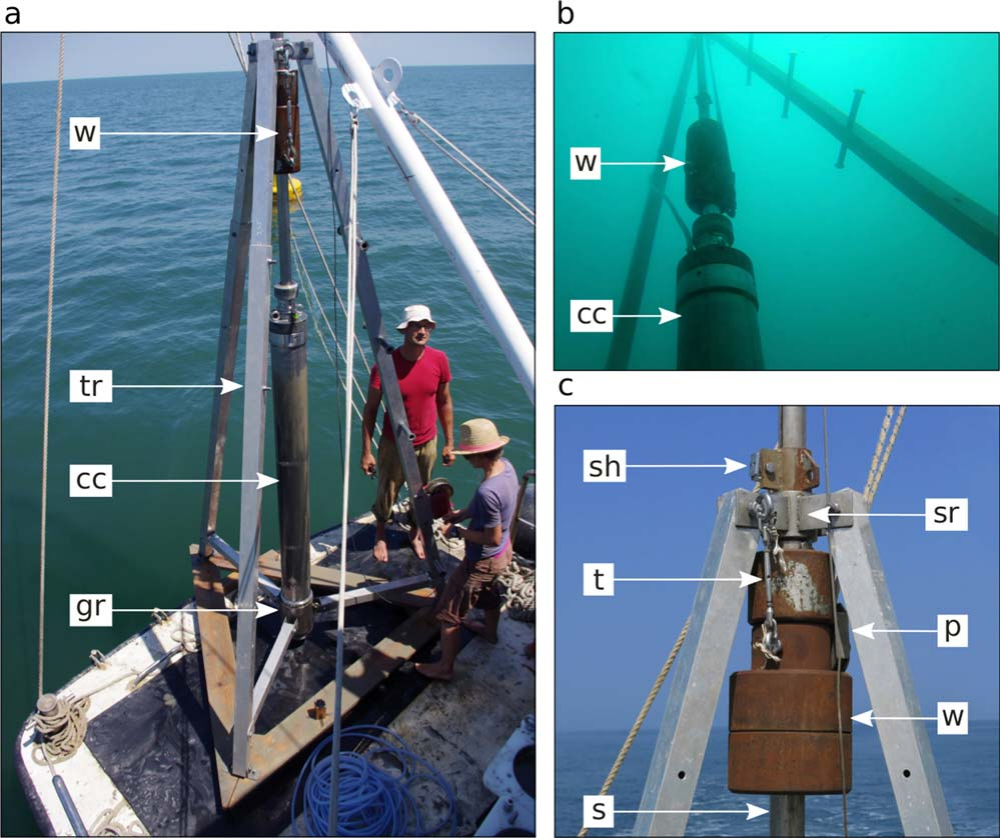
(**a**) The piston corer prior to deployment on the vessel's working platform; cc: coring cylinder; gr: guiding ring; tr: tripod; w: hammering weights. (**b**) Underwater view of the coring cylinder with the hammering weights in action. (**c**) Top part of the tripod with the secured weights; p: pulley (suspension point for the whole corer); s: slit‐rod; sh: slit‐head with latch; sr: slit‐rod guiding ring; t: turnbuckle (weight lock).

**Figure 2:**
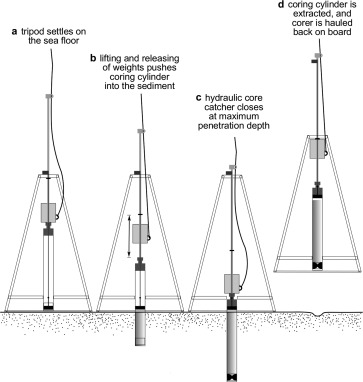
Diagram illustrating the main phases of corer deployment.

The working principle of the newly designed device is that of a piston corer equipped with a percussion system, a hydraulic core catcher, and a stabilizing tripod that keeps the corer in an upright position when lowered to the seafloor (Fig. 1). Coring starts after the tripod has settled on the seafloor and tension is removed from the lifting wire (Fig. 2a). At this stage, the cutting edge of the coring tube is about ten centimetres above the sediment (this distance can be increased to counteract over‐penetration in very soft sediments due to a sinking‐in of the tripod), and the piston is level with the cutting edge, secured in this position by the piston rod. Lifting and releasing of the hammering weights pushes the steel coring cylinder with PVC liner steadily into the sediment, while the piston stays in place (Fig. 2b). Shortly before reaching the desired penetration depth of 1.5 m, the water volume remaining between piston and corer head is prevented from being drained outside and is instead deviated into the small interspace between steel tube and liner (ring‐space). Further corer penetration presses this water down into the rubber sleeve of the core catcher forcing it to close. In its final position, the piston shaft, now fully inserted in the corer head, is blocked by locking jaws, and the rubber sleeve in the core catcher is filled with water and tightly closed (Fig. 2c). The corer can now be extracted from the sediment and hauled on board (Fig. 2d).

### Corer design and main components

#### Tripod

**Figure 3:**
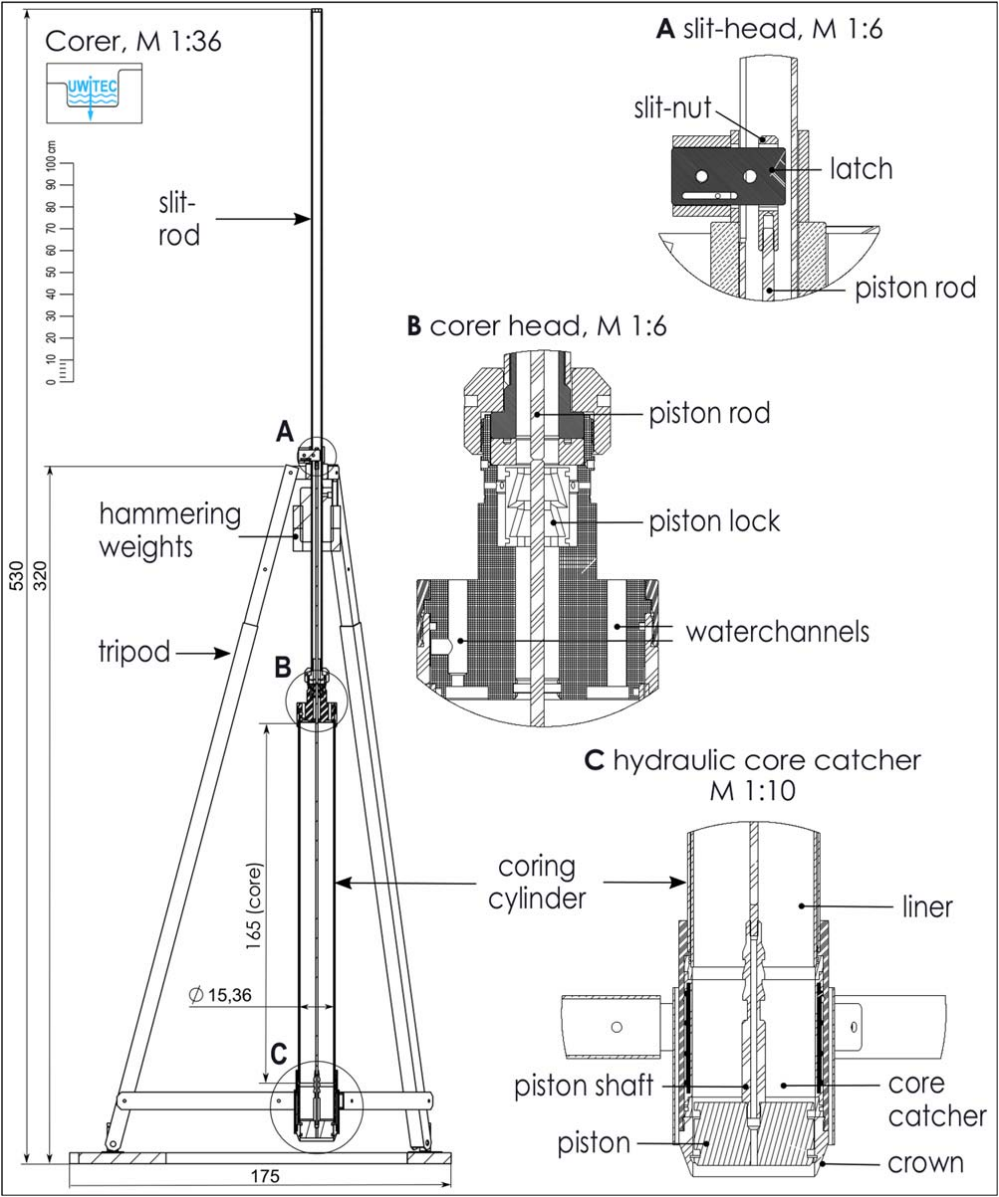
Sketch of the corer with detail view of slit‐head, corer head and hydraulic core catcher.

**Figure 4:**
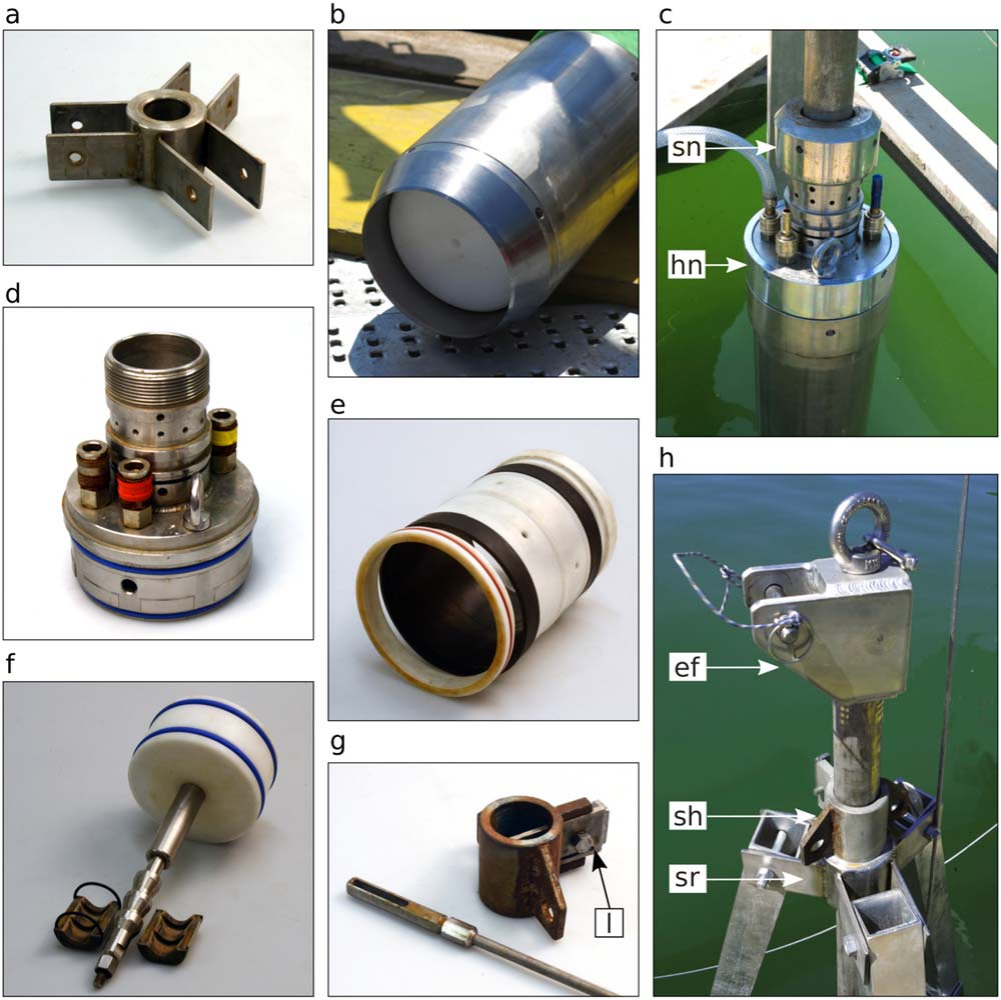
(**a**) Slit‐rod guiding ring with brackets, forming the apex of the tripod. (**b**) Lower part of coring cylinder with cutting crown, piston visible inside. (**c**) Upper part of coring cylinder with mounted corer head and connection to the slit rod; head valves with bleeding nipples and inserted water hose; hn: large head nut; sn: striker nut. (**d**) Corer head. (**e**) Hydraulic corer catcher. (**f**) Piston with piston shaft and locking jaws. (**g**) Slit‐head with latch (l), and terminal part of upper piston rod with slit nut; l: latch. (**h**) View of tripod apex at final penetration depth; ef: slit‐rod end fitting; sh: slit‐head; sr: slit‐rod guiding ring.

The tripod (Figs. 1, 3) is made of three square aluminium bars or “legs” (edge length 7 cm) 3.25 m long, each consisting of two nestable parts for easier transportability. On top, these bars connect to the slit‐rod guiding ring (Figs. 1c, 4a, h), on the bottom to three wooden boards (width 20 cm, outer edge length 2 m) that are cut to form an equilateral triangle resting on the ground. Three horizontal crossbars, each one fixed at a tripod leg near the ground, point toward the central axis where they hold a guiding ring that accepts the coring cylinder and keeps it in vertical position during coring (Fig. 1a). One of the tripod legs bears screw holes for screw‐on steps to climb the tripod during liner extraction and corer re‐assembly. A small manual winch is mounted on this leg: it helps better handle the steel coring cylinder during several phases of corer assembly and dismantling.

#### Core tube unit

The core tube unit consists of the following functional subunits: (1) steel coring cylinder with liner, (2) corer top piece or corer “head,” (3) hydraulic core catcher, (4) piston with piston rod and slit‐rod, and (5) mobile hammering weights.

(1) Coring cylinder This massive stainless steel tube is 1.97 m long and has an outer diameter of 16.5 cm (Figs. 1a, 3). The uppermost portion of the tube is slightly enlarged to form a seating for the corer head that, once inserted, is fixed in its position by a large nut (Fig. 4c). At the bottom end of the cylinder, a 26‐cm‐long section is also enlarged (diameter 19.6 cm) to host the hydraulic core catcher. The cutting edge of the tube, the “crown,” can be unscrewed to insert liner and hydraulic core catcher during assemblage (Fig. 4b). Liners are made of translucent PVC or acrylic glass. The PVC version we used had a wall thickness of 3.7 mm and an outer diameter of 16 cm. The liner length (exactly 167 cm) guarantees a perfect alignment with core catcher and corer head in the assembled system. When storing the steel cylinder, the large terminal head‐nut and the crown should always be mounted to protect the threads from accidental damage.

(2) Corer head The corer top piece or head (Fig. 3 detail B, Fig. 4c, d) is a massive stainless steel lock for the coring cylinder. It is equipped with three valves and several bores that channel the water flow from inside the cylinder during coring. A large bore through the central axis of the head accepts the piston shaft shortly before the corer reaches final penetration depth and blocks the piston in its end position by means of locking jaws (Fig. 3 detail B and Fig. 4f). The head also absorbs and transmits the momentum dealt by the falling hammering weights.

The three valves have different functions: the valve without marking connects to the space above the piston, inside the liner (liner‐space); the red valve communicates with the small interspace between liner and steel tube (ring‐space); the yellow valve accesses both the liner and the ring space depending on the position of the incorporated valve ball, which invariably closes one passage or the other. The mounted valves can accept a hose that, on its other end, has a fitting for a water pump and may assume several functions: a) help fill liner‐ and ring‐space with water immediately after submerging the core tube unit, purging any remaining air from the system; b) signal the final penetration depth. With the hose mounted on the yellow valve during coring, the water that in the last phase of corer penetration is normally pressed into the ring‐space to close the hydraulic core catcher, is now pressed into the hose and can be caught in a bottle at the surface; this water has a known volume (1.7 L), and is therefore an indicator of maximum penetration depth; c) the hose is then used to close the hydraulic core catcher deliberately; by connecting it to the water pump, water is pressed into the ring‐space forcing the rubber sleeve of the core catcher to close; complete closure is signalled by rising pressure in the hose, readable from a connected manometer. When operating the corer in greater water depths that make the use of a hose impractical, an increased driving weight or mounting a special hydraulic cylinder is recommended to ensure perfect closure of the core catcher (see sections “Assessment” and “Comments and recommendations”).

(3) Hydraulic core catcher The hydraulic core catcher (Fig. 3 detail C and Fig. 4e) consists of four parts: a) an adapter ring with O‐ring seals fitting on the lower end of the liner, b) a cylindrical polyacetal support for the rubber sleeve with perforated walls through which the water from the ring‐space passes to close the sleeve in the final coring phase; c) the rubber sleeve itself, secured to the support by means of an upper and a lower collar; d) a terminal protective ring with outer O‐ring seal. The assembled core catcher is 20.7 cm long, has exactly the same inner diameter as the PVC liner tubes (15.7 cm) and is accommodated in the enlarged terminal section of the steel coring cylinder, fixed in its position by the mounted crown (Fig. 4b). The functioning of the hydraulic core catcher is explained in the paragraph “working principle” above.

(4) Piston, piston rod and slit‐rod The piston is made of a cylindrical portion of massive white polyacetal 8 cm high and 15 cm wide. An upper and a lower groove accommodate two thick O‐rings to provide a good fit inside the liner. The stainless steel piston shaft (23 cm high, 2.9 cm diameter), has a threaded lower terminal and is thus removable from the piston body. Its upper portion is milled into a “Christmas tree” shape fitting into the locking jaws in the corer head that block the piston in its end position (Fig. 4f). The shaft is hollow; its upper terminal is also threaded to accept a bolt that connects to the piston rod and can be unscrewed in case the system must be ventilated.

The piston rod is divided into a lower (176 cm) and upper part (108 cm). During assemblage, both parts are interconnected by a special nut. On the terminal part of the upper piston rod, a long nut with an axial slit is mounted to accommodate a latch linking the piston rod to the so‐called slit‐head (Fig. 3 detail A and Fig. 4g). In assembled configuration, the slit‐head rests immediately above the slit‐rod guiding ring of the tripod keeping the piston in the right position throughout the coring process (Figs. 1c, 4h).

The slit‐rod is a heavy, massive 3.1‐m‐long metal tube (Fig. 3). It is connected to the corer head by a striker nut with an underlying striker ring that absorbs and transmits the momentum from the hammering weights to the corer head and the core tube unit (Fig. 4c). The lower part of the slit‐rod acts as a sliding axis for the mobile weights. A stout metal ring bolted into the slit‐rod at about 1 m from its lower end delimits their lifting height. From this point upward, the rod features a continuous longitudinal slit up to the top. Through this slit, the slit‐head with its latch connects to the terminal end of the piston rod. Thus, during coring, when the whole core tube unit penetrates stepwise with every blow of the weights, the slit‐rod can pass by the latch of the slit‐head whose position remains unchanged on top of the tripod. An end fitting mounted on the upper end of the slit‐rod serves as a cable guide for the carrying rope and as a suspension point for the deflection pulley of the small manual winch attached to one of the tripod legs (Fig. 4h).

(5) Hammering weights In the configuration we mostly used during our field work, the piston corer was equipped with four weight blocks of 70 kg in total. A heavy‐duty pulley bolted to the central weight cylinder is the suspension point for the whole coring device. Using a pulley here to create a tackle facilitates corer handling, especially when no high‐power winches for corer deployment and retrieval are available. If a strong winch is available, a robust eye bolt can replace the pulley to hook in the suspension cable. A smaller eye bolt mounted on the same weight block serves to secure the weights on the tripod with a turnbuckle when the corer rests on board (Fig. 1c).

### Extruding system

The extruding system used to extract the sediment from the liner directly on board was specifically designed and developed for the piston corer. The device consists of only a few components, enabling easier transport and handling. It uses hydraulic pressure to extrude the sediment and requires only a simple water pump as installed on almost every working boat. Alternatively, a 12‐volt automatic water pressure pump (e.g., JABSCO™ Par‐Max 2.9) as available in most nautical stores can be operated with the boat battery. The water pressure supplied by such small pumps (2–3 bars) is sufficient to elevate a mass corresponding to the 1.5‐m‐long sediment column inside the liner.

**Figure 5:**
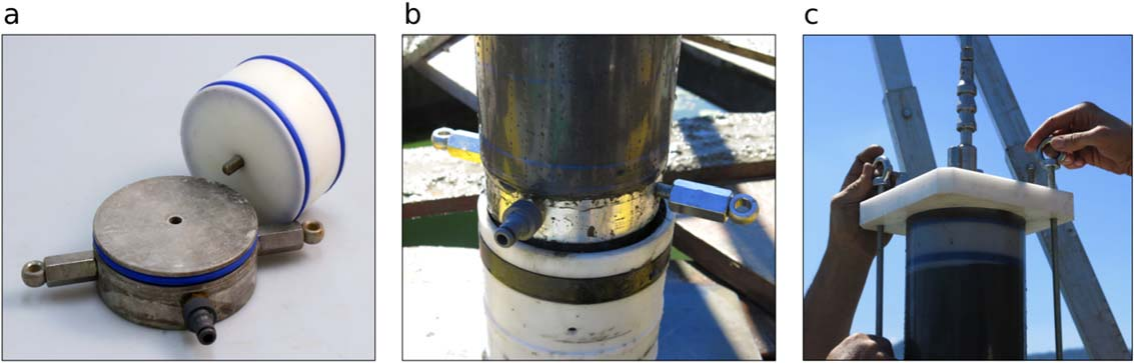
Core extruding system. (**a**) Aluminium base and extruding piston; (**b**) extruding system with superimposed liner; (**c**) liner top with polyacetal collar.

The main components of the extruding system are a cylindrical aluminium base and a polyacetal piston fitting exactly inside the liner tube (Fig. 5a). The aluminium base has a threaded connection for a water hose and two other threaded bores for eyebolts, offset by 90 degrees. The extruding piston resembles the coring piston. It is set directly onto the aluminium base, with a pivot in its lower surface engaging in the central water inlet of the base, thus ensuring a perfectly aligned and stable position of the two elements. During corer disassembly (see below), the liner is placed over the piston‐aluminium base system and the piston is fully inserted until the liner rests on a small rim of the base (Fig. 5b). Then a quadrangular polyacetal collar is mounted on the top end of the liner. Two long threaded rods are now inserted into the eyebolts of the aluminium base and through the holes at the corners of the PVC collar, and fastened with wing nuts (Fig. 5c). This firmly locks the liner onto the basis. By attaching the water hose and using a simple shut‐off valve, the water pressure that pushes the extruding piston slowly upward through the liner can be finely dosed.

## Operation and handling

The corer should be operated by two to three persons. The duration of the whole procedure, including deployment from board, retrieval, liner extraction and re‐assembly, depends on the type of sediment sampled. Soft and wet sediments (silt and clay) are the easiest and fastest to sample. At water depths down to 50 m, a core can be recovered and prepared for slicing in 90 min, with the device ready for re‐deployment. In sandier sediments, more time is needed for the penetration phase, especially when using our “lightweight” configuration. Very firm clays are also hard to penetrate and may require more time to achieve the desired core length of 1.5 m (see Table 1 for details on corer performance).

**Table 1:** Performance data for UWITEC piston corer with hammer action, for both 160 mm and 90 mm liner tubes.

Station	Water depth [m]	Sediment type	Core lengths [cm]	Actual vs. (planned) sampling attempts for core Ø		Mean ratio btw. actual and target core length for core Ø		Number of cores with max. length for core Ø		Percussion method	Dead weights [kg]	Sampling time [min]				
												Corer deployment and retrieval	Percussion time for core Ø		Liner extraction and re‐assembly for core Ø	
				90 mm	160 mm	90 mm	160 mm	90 mm	160 mm			90 & 160 mm	90 mm	160 mm	90 mm	160 mm
Piran	22	muddy sand	144–157	5 (4)	2 (2)	0.91	1	3	2	Manual^a^	40	10–15	15–20	20–30	45	65–80
Isonzo 1	4	sand	133–155	4 (3)	0 (2)	0.95	x	3	x	Manual	40	10	40–60	x	45	x
Venice 1	23	sand, firm clay	100	4 (4)	0 (0)	0.65	x	0	x	Manual	40	15	80	x	45	x
Po 1	21	mud	148–153	3 (3)	3(2)^b^	0.99	1	2	2	Manual	40	10	1–5	5–10	45	60–75
Po 2	21	mud	152–158	3 (3)	2 (2)	0.92	1	1	2	Manual	40	10	1–5	5–10	45	60–75
Isonzo 2	3	sand, muddy sand	155	1 (1)^c^	0 (0)	1	x	1	x	Winch	70	10	15	x	45	x
Piran 2	23	muddy sand	150–163	3 (3)	2 (2)	1	1	3	2	Winch	70	10–15	10–15	10–20	45	65–80
Venice 2	21	sand, firm clay	27–159	5 (4)	0 (0)	0.75	x	3	x	Winch	70	10–20	30–60^d^	x	45	x
Venice 3	21	sand, firm clay	73–159	4 (4)	0 (0)	0.63	x	0	x	Winch	70	10–20	30–60	x	45	x
Brijuni	44	muddy sand, palaeosoil	86–162^e^	6 (4)	0 (0)	0.88	x	4	x	Winch	70	10–15	15–20	x	45	x
Porec	31	muddy sand, firm clay, palaeosoil	150–165	5 (4)^f^	0 (0)	0.91	x	3	x	Winch	70	10–15	15–30	x	45	x
Panzano	13	mud	150–159	3 (3)	2 (2)	1	1	3	2	winch	70	10	1–5	5–10	45	60–75

a Hammering by hand may include some resting time between cycles.

b None of the cores reached target length, 3 cores lost, only 1 retrieved.

c Test coring drive for increased dead weight and winch supported percussion.

d Long percussion times due to difficult penetration in firm clay layers.

e Coring aborted due to hardly penetrable firm clay layers of terrestrial origin.

f 1 core lost due to defective liner tube; two coring drives aborted because of drifting boat.

### Deploying the corer

The piston corer can be operated either from specific working platforms (mainly used on lakes or rivers) or from vessels with a working area of at least 5 m^2^ where the tripod can rest and the corer be assembled and disassembled comfortably. Ideally, this platform should enable short‐term storing of the extracted liners in an upright position and performing the slicing process. Additionally, an A‐frame or beam with winch or a crane of at least 3.5 m height is required. The winch available on our supporting vessel was an anchor winch (Lofrans' Falcon Horizontal Windlass) with a max. lift load of 850 kg. With a pulley tackle, however, much weaker winches can handle the corer. A high lift load (either using tackles or a powerful winch) is advantageous especially for extracting the corer from tougher substrates (sands, compact clays) to overcome increased friction or vacuum effects that may create in the hole under the corer. Vessels with the described equipment are commonly available at marine biology stations or can be rented at most port towns.

Before the coring operation starts, the supporting vessel should be stabilized by at least two anchors. This is especially important for work at sea, where currents may change quickly and unpredictably and the boat might drift away from the vertical axis above the corer within minutes if only a single anchor is used. A deeply inserted corer, connected to the drifting boat by an increasingly oblique suspension cable, can become an irremovable anchor itself and a source of major troubles (USEPA 1999).

Care must be taken that the piston remains in its position level with the cutting edge of the coring cylinder during immersion. A piston accidentally pushed upward by the entering water will result in shorter cores. Here, it helps to fix the upper end of the piston rod (the slit‐head) to the tripod with a short rope that is removed before the corer disappears completely under water. The inner spaces of the core tube unit are flushed by plugging venting nipples into all valves of the corer head and keeping the corer submerged on the surface until all air has escaped. Alternatively, at this stage, liner‐ and especially ring‐space can also be flushed manually by attaching the water hose to the corresponding corer‐head valves (Fig. 4c, d) and filling the inner volumes completely. Finally, the water hose is connected to the yellow valve (accessing both liner and ring space), the venting nipples, the manual winch and the turnbuckle securing the mobile weights are removed, and the device is now ready to be lowered gently to the seafloor avoiding accidental elevation of the piston by excessive sinking speed. Touchdown is signalled by a tension drop on the suspension wire or, if available, indicated on the traction force display of the winch. The water hose is now disconnected from the pump and its end placed in a measuring bottle to catch the “rubber sleeve water” displaced by the piston before reaching final penetration depth (see chapter “corer head”). On muddy soft bottoms, the core tube may penetrate the sediment simply due to its own weight and that of the hammering blocks. With coarser grain size and/or firmer sediment, however, the percussion mechanism is used to achieve the desired core length. Hammering can be performed manually by grabbing the suspension cable and lifting and dropping the weights repeatedly, but using the winch is recommended and becomes necessary if the weights are substantially increased. For deep‐water coring, a heavy driving weight could replace the lighter hammering blocks, reducing or potentially eliminating the hammering phase (see section “Comments and recommendations”). The risk of pulling the corer off the sea floor by lifting the weights too high is practically non‐existent when manually hammering, and winch‐supported hammering also conveys a good “feel” for the appropriate drop height of the blocks by inspecting the line length hauled for every blow. At intermediate depths down to 50 m, the length of the suspension cable is also effective in decoupling minor wave‐induced motions of the vessel (Glew et al. 2001). When complete penetration is signalled by the 1.7 L of water caught in the measuring bottle, the water hose is reconnected to the water pump and the rubber sleeve of the core catcher is hydraulically inflated. A pressure rise to 1.5–2 bars indicates sleeve closure, and the corer can now be extracted from the sediment and pulled aboard. Before placing it on the working platform (using some square timbers as pads), the deflection pulley of the manual winch is hooked onto the end fitting of the slit‐rod and the turnbuckle mounted to block the hammering weights.

### Extracting the liner

**Figure 6:**
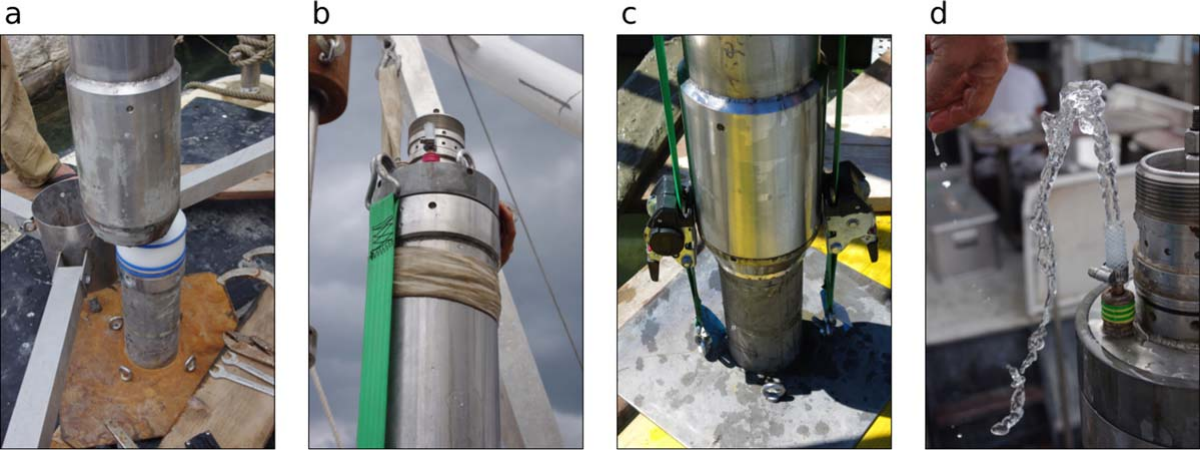
Liner extraction procedure. (**a**) Placing cylinder on pedestal over extruding system; (**b** and **c**) applying lashing straps to pull cylinder down on aluminium basis; (**d**) inserting bleeding nipples into head valves to drain water from hydraulic core catcher.

Once the corer stands firmly on the platform, the manual winch is mounted and the coring cylinder secured with a round sling. Now the striker nut connecting the cylinder to the slit‐rod is loosened, and by lowering the cylinder slightly, the coupling nut between upper and lower piston rod becomes accessible and can also be unscrewed. The coring cylinder is thus detached from the tripod system, and using the manual winch, it can be carefully manoeuvred out of the guiding ring and onto the pedestal with the extruding system, which is set up very close to the guiding ring (Fig. 6a). In the next steps, the valves of the corer head are ventilated by inserting venting nipples, and the cylinder is pulled down onto the extruding piston and the aluminium base using a pair of lashing straps (Fig. 6b, c). This will force the water entrapped in the core catcher to flow out through the head valves and the cylinder to slide down over the extruding piston and the aluminium base (Fig. 6d). Now the corer head can be removed, the cutting crown of the cylinder unscrewed (the core catcher will then slip out onto the pedestal) and the cylinder itself pulled over the liner using the manual winch. During this process, the liner must remain stable, upright and well set on the aluminium base of the extruding system and not accidentally be lifted from it together with the steel cylinder. Finally, the liner, now standing free on the pedestal, is cleaned of sediment and fitted with the polyacetal collar and the locking rods, as described in the section “extruding system.” It is now ready to be moved off the pedestal and stored in a secure place on board where sediment slicing can take place.

### Extruding and slicing the sediment

**Figure 7:**
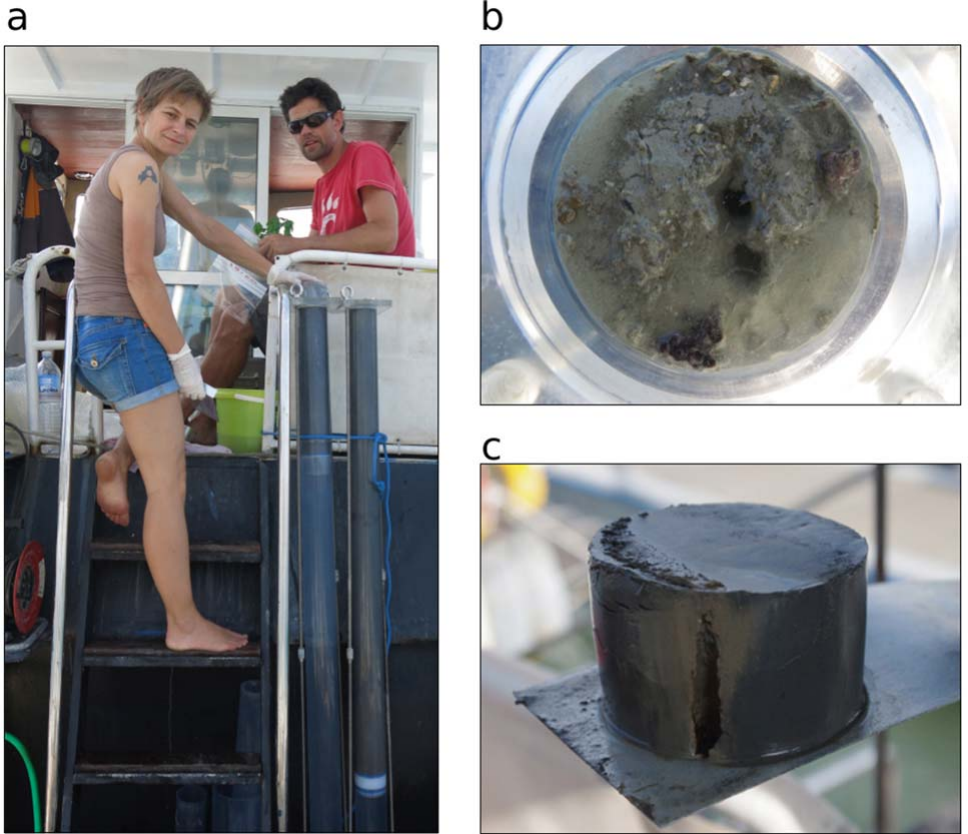
(**a**) Slicing setup at a floor level difference between working platform and boat deck, allowing comfortable working position and close visual control of sediment extrusion; (**b**) Undisturbed surface of a core taken at the Venice station (sandy sediment); (**c**) subsample from a Po‐Delta core, mud, with section of infaunal burrow.

The 1.5 m long cores are sliced in upright position. The person cutting the sediment should therefore be able to sit comfortably level with the upper end of the liner during slicing. Either a stepladder can be used, or the liner can be positioned such that a floor level difference allows for easy access to the liner top (Fig. 7a).

Usually, the sediment surface inside the liner is covered by water. This water can be sampled using a syringe before slicing (Fig. 7b). Then, by connecting the water hose to the aluminium base of the extruding system and regulating the shut off valve as required, the piston is pushed slowly up the liner, extruding the sediment at the upper end. The thickness of the sediment to be sliced off is adjustable to the millimetre. This accuracy, however, is compromised if air enters the space between aluminium base and extruding piston. An air bubble here functions as a shock absorber and should be avoided. To cut the extruded sediment, a thin metal sheet or a spatula is used combined with liner segments of specific height (1 cm, 2 cm, 5 cm etc.; Fig. 7c); these segments act as spacing rings for the desired cutting intervals.

### Core archiving and splitting

Cores do not necessarily need to be fully sliced directly after coring, but may be prepared for storage or for a different sampling approach such as lengthwise splitting, with one half of the core (the archive half) being used for sedimentological and stratigraphic description, and the other (working) half for discrete sampling. In this case, the liner is extracted and treated the same way as for immediate slicing. Using the extruding system, the sediment column is pushed upward until its surface is about 5–7 cm from the upper rim of the PVC tube. The overlying water is removed with a syringe, and the remaining empty space filled with a block of floral foam, which is simply pushed over the tube until it touches the sediment surface. This stabilizes the sediment surface and absorbs residual water. An appropriate rubber cap is then applied over the tube end and fixed with reinforced tissue band. The liner can now be laid down and sealed at the lower end. A tube cutter tool is used to remove the excess portion of liner by cutting exactly between extruding piston and sediment. A rubber cap and, if necessary, a thin layer of floral foam again provide sealing and sediment stabilization. Liners prepared this way can readily be moved around and tilted without compromising the original stratigraphic structure of the enclosed sediment column.

## Assessment

Our several‐weeks‐long coring operations in summer 2013 and 2014 yielded more than 50 cores. Additionally to the 160‐mm‐diameter core tube unit, we used a thinner unit with an outer liner diameter of 90 mm. Interchangeable core tube units of different diameter represent important extensions of our piston corer (see section “Comments and recommendations”). The thinner coring cylinder was applied in stiff sediments where the 160‐mm device reached its limits with the weight configuration used during our fieldwork. Sampling depths ranged from three to 44 m, and a full range of sedimentary environments were targeted, from almost pure sands several miles off the Venice lagoon to muddy bottoms off the Po river delta.

### Performance across different sediment types

**Figure 8:**
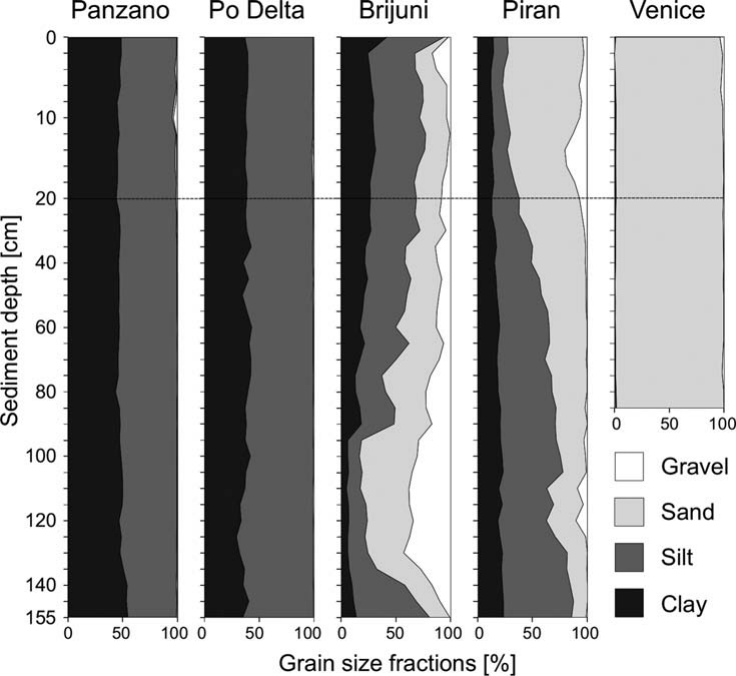
Particle size distributions at the five sampling areas Panzano Bay, Po Delta, Brijuni Islands, off Piran and off Venice. Grain size categories follow DIN 4022.

Figure 8 shows particle sizes at the five sampling areas Panzano Bay, Po Delta, Brijuni Islands, Piran and Venice. The down‐core grain size variation was negligible at stations in Panzano Bay, on the Po Delta, and at theVenice lagoon, but substantial at the Brijuni and Piran stations. The Brijuni sediments are characterized by gravelly sediments near the bases of the cores, made up mainly of bryozoan skeleton parts and, to a lesser extent, larger molluscan shell fragments. Some of the Venice and Brijuni cores showed sediments of clearly terrigenous origin such as dry and firm fluviale clays (Venice) and peat (Brijuni) at the base. This stratigraphy indicates that these cores covered the whole Holocene transgression event. Occasionally, at the Venice station, this terrestrial‐to‐marine transition is recorded at core depths as shallow as 20 cm. Both clay and peat layers were difficult to penetrate with our corer, requiring lengthy hammering times to reach target depth or, in a few cases, an early abortion of the coring drive. Interestingly, these stations were characterized by a paleo‐relief that caused the marine‐terrestrial sediment interface (the Holocene transgression boundary) to vary considerably in depth between closely adjacent cores. This shows how problematic pooling of multiple cores from the same area can be, and again highlights the advantages of large diameter cores over small ones: many analyses can be performed on a single coherent sediment sample instead of splitting them among several cores of uncertain correlation.

Table 1 summarizes corer performance data for all coring stations, for both core‐tube‐unit configurations (160 mm and 90‐mm liner diameter) and for two different types of percussion methods (40‐kg weight block, manual lifting vs. 70‐kg block, winch‐supported lifting).

An additional sampling area—Isonzo—is listed but not included in Fig. 8. The stations there are located close to the Isonzo river mouth and characterized by sandy‐gravelly sediments and very shallow water (3–4 m). They were mainly sampled to test the corer under easily controllable conditions and to assess its performance in sandy environments. These tests convinced us to refrain from further using the 160‐mm core tube unit in pure sands because penetration proved to be very difficult.

Target core lengths mentioned in Table 1 are: 160 cm for the 90‐mm diameter and 150 cm for the 160‐mm diameter. The mean ratio between actual and target core length is a measure for core shortening only when full penetration of the corer was achieved. At muddy or muddy‐sandy stations (Po, Panzano, Piran), the ratio is 1 or close to 1, i.e., core shortening is minimal or absent. At the other stations (Venice, Brijuni), the considerably smaller mean values are not due to excessive core shortening but to the abortion of coring drives (impenetrable sediment layers, mostly dry, firm and stiff clay), resulting in cores much shorter than target length.

### Operation times

The time needed for deployment (lifting the corer over board and lowering it to the seabed) and retrieval (extraction from the sediment, hauling on board and repositioning on working platform for disassembly and liner extraction) varies with water depth and sediment properties (Table 1). At moderate depths, lowering and lifting the corer were always quick, but extraction from stiff clays (Venice) sometimes had to be performed slowly and carefully to avoid damaging the equipment due to high tractive forces. Percussion times were very short in muddy sediments (1–5 min at Po and Panzano), increased with higher sand content (10–20 min at Piran) and peaked at 80 min (manual percussion) in compact clays (Venice, Table 1). The latter are extremely difficult to penetrate with most types of corers (Glew et al. 2001), yet we still produced a few cores that successfully cut through > 1‐m‐thick clay layers. Since these (terrestrial) layers were not relevant to our investigations, we aborted coring when encountering the “clay resistance,” thus saving precious time.

The time needed for liner extraction and corer reassembly is considerably shorter for the 90‐mm coring tube configuration (about 45 min) vs. the thicker setup (60–80 min, Table 1). The handling times increase both due to heavier equipment and when sampling sandier sediments (stronger friction may slow down the positioning of the extracted liner over the extruding piston and pedestal).

### Core shortening

Performance data across a broad range of sediment types show that the corer in its larger configuration (160‐mm‐diameter coring tube) works well in mud and sandy mud but reaches its limits in sediments with a high sand content or in firm, compact clays. The 90‐mm coring tube provides cores even from the toughest sediments albeit taking time. The performance data also show that in the coring drives reaching target depth, core shortening is minimal or absent. This is an important result because core shortening is a frequent drawback associated with many coring devices, especially gravity corers. Core shortening (also termed “core compaction” or “entry deficit”) describes a phenomenon in which the length of the sediment column sampled is shorter than the corer's penetration depth. This is not due to sediment compression during coring, as erroneously assumed for some time. Rather, sediment is lost by being pushed laterally outward ahead of the cutting edge of the corer due to frictional forces inside the tube, which increase with sediment depth (see e.g., Glew et al. 2001 for a detailed description). Three main factors minimize this undesirable effect in our coring device:

1. *The presence of a piston*; the closely fitting piston in the sampling tube creates a vacuum between piston and sediment surface during penetration; this counteracts the frictional forces that, in a configuration with open barrel, would cause the core to be drawn down with the tube. Sediments are “sucked” evenly and undisturbed into the sampling tube, sample entry is facilitated and core shortening reduced or almost eliminated (Blomqvist 1991; Nesje 1992; Mudroch and MacKnight 1994; Glew et al. 2001).
2. *Large core diameter*; small coring tubes, although easier to push, encounter excessive internal friction in the core, and recovery may be incomplete (Glew et al. 2001). Large‐diameter coring tubes minimize wall friction, down‐bowing of sediments along the tube walls and core shortening, considerably improving core quality (Pedersen 1985; Blomqvist 1991; Chaney and Almagor 2015).
3. *Slow entry speed*; most gravity or piston corers gain their penetration energy from a free fall starting at a considerable distance above ground, which leads to a fast penetration. Our corer is provided with a supporting tripod that is gently lowered to the sea bottom; only after landing does the coring tube start sinking into the sediment from a pre‐set height of about 5–10 cm above the sediment surface and is then pushed further down by the hammering weights.

### Sediment‐water interface

The corer's slow penetration velocity not only decreases the risk of core shortening (Blomqvist 1991) but also prevents the formation of a bow wave in front of the corer's cutting edge, which can wash away surficial sediment layers before impact (McIntyre 1971; Glew et al. 2001). Accordingly, we never observed a disturbed sediment‐water interface in the retrieved cores. Here, careless handling during liner extraction and storage could do much more harm than the coring process itself. Our results demonstrate that this sampler largely preserves the integrity of in‐situ sediment properties such as shear strength, bulk density and bedding, and also maintains an intact sediment‐water interface.

### Comparison with other corer types

No one type of sediment corer is applicable to all types of studies and conditions (Glew et al. 2001). However, by comparing our corer with a range of other corer types presently used for sampling limnic or marine sediments, we would like to stress those features that represent improvements over existing devices and make this corer a widely usable instrument. Table 2 summarizes key specifications along with advantages and possible design shortcomings. The list includes box corers, gravity corers, Kasten‐corers, Slo‐corers, multicorers, piston corers, percussion corers and vibracorers that are comparable to our corer in dimensions, weight or core diameter used. Small, simple hand‐held corers and very large and complex instruments such as deep‐sea drill corers are not considered.

**Table 2:** Comparison between different corer types commonly used on smaller research vessels or platforms, listing key technical specifications and respective advantages and disadvantages. Grey shading highlights the corer described in this article. References provided as footnotes at the end of the table.

Corer type, dimensions	Liner and (penetration) length [cm]	Working depth [m]; Sediment	Corer weight, (dead weights) [kg]	Core catcher/Vertical extruding system	Infrastructure	Advantages	Disadvantages	Ref
Ocean instruments BX 610 **Box Corer** 20 x 30 cm	60	Full ocean depth?;soft or unconsolidated	520	Yes / ?	Medium vessel, crane and winch, 2 operators	• Large sample volume • undisturbed surface sample • intact subsampling possible	• Limited penetration depth	1
Wildco® Kajak‐Brinkhurst Heavy **Gravity Corer** 5 cm ID	51 to 244	300;soft, fine‐grained	50 (max. 26)	Yes / no	Small vessel, crane and winch	• Eggshell core catcher • complete flow‐through sampler (limits shock wave) • light, versatile and easy to handle	• Core shortening • small core diameter	2
Wildco® Ballchek **Gravity Corer** 5 cm ID	51 to 244	300;loosely consolidated, finegrained	20 (14)	Yes / no	Small vessel, crane and winch	• Sediment only in contact with inert plastic • eggshell core catcher and check valve sealing mechanism • light, versatile, easy to handle	• Shock wave may disturb surficial sediment layers • core shortening • small core diameter	3
Carma® **Gravity Corer** SW‐104 10.4 cm ID	65.4 (50) to 200 (185)	Tested to 1000;clayey to slightly sandy	100 (17 to 100)	Yes / ?	Small to medium vessel, crane and winch	• Core catcher and head valve for reliable sample retention • mechanical diaphragm type core catcher	• Core shortening • shock wave may disturb surficial sediment layers • limited penetration depth in stiffer sediments (clay, silt) • may not penetrate vertically?	4, 5
AWI **Kasten Corer** 30 x 30 cm	600 1200 1800	Full ocean depth?;soft, muddy	NA (max. 3000)	Yes / no	Large vessel, 2 powerful cranes and winches, handling system for long cores, specialist staff	• Large sample volume • intact subsampling and analyses of scarse biogenic components • low core shortening and disturbance	• Restricted to fine sediments • requires large and expensive infrastructure • use expensive • long cores must be lifted to a horizontal position and maneuvered over the rail to bring aboard the vessel	6,7, 8
USGS hydraulically damped corer (**Slo‐ corer**) 10.7 cm ID	30 (sand) 60 (mud)	Full ocean depth?;soft, muddy	500 (340)	Yes / ?	Large vessel, crane and powerful winch, heavy‐gauge wire	• Penetrates the sediment slowly in order to minimize disturbance of the water/sediment interface • no shock wave effect • spring‐loaded paddle slides under liner after extraction from sediment	• Limited penetration depth • relatively heavy, requires large vessel	9, 10, 11
KC Denmark **Multi‐Corer** 8 x 10.4 cm ID	80 (50)	6000;soft, muddy	550 (max. 300)	Yes	Large vessel, crane and powerful winch, 2 or more operators	• Multiple samples in a single drive, quick change of tubes, undisturbed surface samples • spring loaded plate closes liner at sediment surface	• Limited penetration depth • relatively heavy therefore requires larger vessel and powerful winch	12
KC Denmark **Piston Corer** (Kullenberg type) 9.9 cm ID	200 to 400	Full ocean depth;soft, muddy	280 (max. 280) + 30 kg release weight	No	Large vessel, crane and winch	• Piston facilitates sample entry and reduces core compaction • piston promotes sample retention	• Possible failure of piston activation • uppermost sediment may be disturbed or lost • no core catcher	13, 14
Carma® **Piston Corer** CP‐20 9.4 cm ID	500 to 3000	Full ocean depth;soft, fine	NA (650 to 1850)	Yes / no	Large vessel, 1 or 2 cranes, handling system for long cores, 1 or 2 powerful winches,specialist staff	• Recovery of undisturbed sediment cores • long cores possible • no water depth limitation	• Requires large and expensive infrastructure • piston and piston position at penetration may fail • disturbed surface layer • heavy and awkward to deploy and recover • long cores must be lifted to a horizontal position for retrieval over vessel rail	15
UWITEC **Piston Corer** with hammer action 9 or 16 cm ID	160 (150) to 300 (290)	Full ocean depth;soft to sandy	220 for 9 cm ID, 250 for 16 cm ID (70, max. 500)	Yes / yes (hydraulic pressure)	Medium vessel, crane and winch 2 operators	• Piston and internal core catcher for better sample retention • hammering weights enable penetration of tough sediments • sediment surface is not disturbed • supporting tripod for vertical entry • large sample volume • upright core extruding system	• Larger configuration operating only in soft to muddy sand sediments • long hammering time in tough sediments • use in rough marine conditions not recommended	
Nesje **Percussion Corer** 6.3 / 11 cm OD	Max. 600	Up to 150; fine to coarse‐grained	100 (20)	Yes / no	Small vessel or raft,jack, 2‐3 operators	• Piston and orange‐peel type core catcher for reliable sample retention • hammering weights enable penetration of tough sediments • light‐weight, easily transportable	• Percussion can cause disturbance to sediments of alternating density or grain size • no supporting stand • in longer cores, lack of steel coring cylinder may lead to vibrations and wobbling of PVC liner during hammering	16, 17
CMS‐Geotech Standard Marine **Vibrocorer** 8.6 cm ID	300 400 500 600	300;soft, loose sands and silts	1800	Yes	Large vessel, crane and powerful winch, heavy‐ gauge wire, 2 or more specialist operators	• Piston and core catcher for better sample retention • preserves sediment stratification	• Core shortening • sensitive to drifting of boat or platform, resulting in tube bending and sample loss • shock wave may disturb surficial sediment layers • labor intense to operate • very heavy, requires large vessel and powerful winch	18
EPA Reference **Vibrocorer** 10 cm ID	450 (sand) 600 (mud)	1200;from clay to packed sands	70	No / no	Medium vessel, crane and powerful winch, 2 operators	• Preserves sediment stratification	• Relatively compressed and shortened core samples • sensitive to drifting of boat or platform, resulting in tube bending and sample loss	19

1) http://oceaninstruments.com/products/multi-corers/mc-400-multi-corer/ (10. 12. 2015).

2) http://www.rickly.com/as/kbcoresampler.htm.

3) http://www.rickly.com/as/ballchek.htm (10. 12. 2015).

4) Magagnoli and Mengoli (2000).

5) http://www.carmacoring.com/products-and-solutions/gravity-corer.html (10. 12. 2015).

6) Parker and Sills (1990).

7) Kögler (1963).

8) https://www.awi.de/forschung/geowissenschaften/marine-geologie/werkzeuge/grossgeraete-auf-see/kasten-corer.html (10. 12. 2015).

9) Jahnke and Knight (1997).

10) Bothner et al. (1997).

11) http://www.whoi.edu/page.do?cid=11258&pid=8415&tid=282 (10. 12. 2015).

12) http://www.kc-denmark.dk/products/sediment-samplers/multi-corer/multi-corer,-8-x-oe110mm.aspx (10. 12. 2015).

13) Mudroch and MacKnight (1994).

14) Glew et al. (2001).

15) http://www.carmacoring.com/products-and-solutions/carma-piston-corer.html (10. 12. 2015).

16) Nesje et al. (1987).

17) Nesje (1992).

18) http://media.wix.com/ugd/43b9e6_ad0bfd6e5dc042c3803c38bc9d82f2dc.pdf (10. 12. 2015).

19) USEPA (1999).

Blomqvist (1991) lists a number of crucial corer features for producing unbiased and undisturbed sediment samples. (1) A supporting stand; this feature guarantees vertical and slow entry of the coring tube (coring starts only after the device settles on the seabed), thus preventing bow wave formation. A stand also helps decouple wave‐ or wind‐induced vessel motions from the coring device. (2) A piston; the most effective approach to reduce core shortening. (3) Large coring‐tube diameter, yielding voluminous samples and minimizing core shortening. (4) An effective core catcher, which secures the sample during extraction and retrieval and does not disturb the sediment as it enters the tube.

Many corer types listed in Table 2 feature one or several of these specifications, but none combines them all. For example, gravity corers are equipped with core catcher and, in the case of the Carma^®^ gravity corer, can provide cores with reasonably large diameters (about 10 cm), but they lack a supporting stand: freefall deployment can create a shock wave disturbing surficial sediment layers upon impact. Likewise, rapid sediment penetration and lack of a piston may promote core shortening in this corer type. The so‐called Slo‐corer is equipped with supporting structure and hydraulic damper: this enables slow and careful entry and penetration, but limits penetration depth. Larger piston corers can produce very long cores, but the lack of a supporting stand increases the risk of tilting and of similar problems as with gravity corers (shock wave formation, core shortening due to rapid penetration). The UWITEC piston corer combines piston, supporting tripod, large core diameter and internal core catcher in a single design, with all the respective benefits (slow entry, undisturbed sediment surface, minimum core shortening, very good sample retention). Moreover, the hammering system guarantees successful coring even in tougher sediments and core lengths beyond those reachable solely using corer inertia as penetration force. The simple and very effective extruding system for upright slicing represents a further advantage. All these features contribute to the instrument's high versatility and the high‐quality cores it can produce.

### Financial aspects

Costs are a crucial aspect when sediment cores are part of the planned fieldwork and the available budget is limited. Table 2 omits purchase costs because most manufacturers make their prices available to potential buyers only upon request and on a confidential basis. The manufacturer of our coring device has provided approximate costs that range from 15 000 to 25 000 € (17 000 to 27 000 $) depending on the required version and specific accessories. This price is lower than that of most other models listed. Importantly, the budget available for coring not only benefits from limited purchase costs, but also from the fact that the corer can be deployed from moderately sized supporting vessels without sophisticated technical equipment, and from dispensing with expensive technical support teams. The few requirements – a winch, a beam or A‐frame and sufficient working space – are typically available on fishing or ordinary working boats in most ports. One day of training by an experienced person is sufficient to gain the necessary expertise enabling researchers to operate the corer successfully by themselves.

Some of the structural and performance aspects of our corer are further assessed in the following notes:

### Large core diameter

The development of this corer was prompted by the need for abundant mollusc shells from individual cores from sediment depths exceeding those of comparable gravity corers. Other sampling methods we initially considered included diver‐operated suction sampling, as performed by Kosnik et al. (2007) in a shallow Great Barrier Reef lagoon. Those researchers sunk a sheet metal tube 56 cm in diameter into the sediment using a vibracore head and then suction‐sampled it by divers. In the northern Adriatic Sea, however, this method proved to be much more expensive, was more difficult to perform in greater water depths, was probably inapplicable on muddy bottoms, and was poorly suited to gain thin and clean subsampling intervals. Even in high sedimentation settings with comparatively low shell densities, the amount of shell material we retrieved with a single 16‐cm‐thick core well represented the local diversity (unpublished data). An important advantage is the simple subsampling of the sediment by slicing. The large amount of sediment this core diameter yields enables multiple simultaneous investigations on one sample, avoiding pooling of material or data from several smaller cores (Burke 1968). The fewer deployments with the large corer diameter are cost and time efficient. On soft sediments, one run, including assembly, coring and liner extraction on board, requires about 90 min (Table 1). The gained material corresponds roughly to that from three equally long cores with a diameter of 9 cm, but the time needed to operate a thinner corer is only slightly less.

### Hydraulic core catcher

The large core diameter requires an effective core catcher to prevent sediment loss during the critical extraction and retrieval phases. Contrary to most other core catcher types, which close only after the coring tube is extracted from the sediment, the UWITEC piston corer features an internal core catcher. This seals the bottom of the core before core tube extraction is initiated, preventing sediment loss already from the onset of the extraction process. While many simpler core retainer models (e.g., spring or “orange‐peel”‐type devices) interfere with the sediment column in the coring tube (Glew et al. 2001), the rubber sleeve of the hydraulic core catcher is perfectly aligned with the liner and does not disturb the sediment as it enters the tube. As an integrated structure, the hydraulic core catcher requires enlarging the lower part of the steel coring cylinder. This increase of the cross sectional area of the coring barrel is small in the coring cylinder for the 9‐mm liners and more pronounced in the thicker barrel for the 160‐mm liners, contributing to the penetration difficulties in very compact sediments. The hydraulic core catcher worked successfully on both coring configurations and in all types of sediments. The water hose used to close the core catcher independently from the closing mechanism of the corer has several advantages at depths down to 100 m. First, the water pressure necessary to close the rubber sleeve (about 2 bars) can be controlled using a manometer, and it can be readjusted if pressure drops. Second, the hose enables closing the sleeve at every stage of coring. Thus, cores shorter than 1.5 m, which otherwise would be lost (the standard closing mechanism of the corer is triggered only after complete penetration), can also be retrieved. In greater water depths, however, handling hundreds of metres of water hose might become cumbersome; here it is preferable to omit the hose and use a considerably larger dead weight to ensure closure of the core catcher via the standard hydraulic closing mechanism.

### Extruding system

The dedicated extruding system is a strong advantage. As Glew et al. (2001) point out, “core extruding and subsampling equipment is as important as the coring devices themselves in as much as this equipment must be capable of taking full advantage of the intact nature of the cored material.” Especially the large and heavy cores produced by the larger coring barrel call for performing the extruding and slicing process directly on board. Our simple and efficient hydraulic device works on the upright core and guarantees accuracy and precision in cutting the sediment. The minor difficulties encountered in very sandy sediments (greater friction on liner wall and partial obstruction of extruding piston) can be solved by more powerful water pumps than those we used.

### Robust and simple design

One strength of the corer is its robust and simple design. It is easily dismountable and transportable. Except for the steel coring cylinder, a single person can carry any individual part; all the components fit into an ordinary van. The manufacturer provides multiple spare parts for those components that are most likely to be worn (e.g., O‐ring seals) or lost during sampling (smaller items such as nuts, bolts, rods, locking mechanisms, tools). This helps avoid sudden and costly sampling interruptions. Many other components can be replaced, fixed or adjusted drawing on standard services and supply stores.

### Operating conditions

The corer works best under calm marine conditions, in shallow water and on muddy sediments. Nonetheless, light swells posed no problems. The corer can also be adapted for much greater depths than those we reached. This, however, would involve heavier weights (see section “comments and recommendations”) to ensure immediate sediment penetration, shorter or eliminated hammering phases, and secure closure of the hydraulic core catcher without using a water hose.

## Comments and recommendations

Sediment coring is used in many scientific disciplines for data collection. Coring costs increase with water depth, variable sediment composition, core length and amount of material needed because these require more sophisticated and heavier coring equipment and correspondingly larger vessels, exceeding the means of many smaller research projects.

The piston corer we introduce herein is an excellent alternative yielding long, undisturbed cores and large sediment volumes at modest costs, with minimal limitations regarding water depth or sediment composition. These key features of the corer, combined with its robustness and ease of use, are an incentive to implement research that would otherwise be foregone for financial or logistic reasons.

Our fieldwork with the UWITEC piston corer led to considerations about extensions, modifications and adaptations that are potentially interesting for future users elsewhere.

### Exchangeable core tube units

A key extension is the possibility of an alternative core tube unit (same length but smaller diameter, e.g., 9 cm). This unit, consisting of corer head, steel cylinder with liner and integrated core catcher like the bigger 16‐cm device, fits to the same tripod and is operated the same way as its larger “brother.” The advantages are faster (by ca. one third) and easier deployment and better penetration in tough sediments. Core sealing and handling is also simplified, because a 9‐cm‐thick core weighs less than one third of an equally long 16‐cm thick core.

### Heavy‐weight configuration for deep‐water coring

Besides a strong winch, the use of more and larger weight blocks requires a prolongation of the tripod legs and, as a consequence, also a length adjustment of slit‐rod and piston‐rod in order to extend the apex area of the construction where the blocks are accommodated. According to the manufacturer, this modification is easy to implement and would allow weights of up to several hundred kilos. In such a configuration, the piston corer works almost like a gravity corer, penetrating even tougher sediments either by its own weight or with only a few blows of the heavy hammer. Moreover, the heavy weights ensure closure of the hydraulic core catcher at final penetration depth without using a water hose. Alternatively, the corer could also be provided with a special hydraulic cylinder that is triggered when core tube extraction starts, injecting the sleeve‐closing water into the core catcher.

### Increased core diameter and core length

In principle, also core tube units with diameters > 160 mm can be realized. This, however, requires an enlargement of the internal hydraulic core catcher with a consequent increase of the cross sectional area of the coring barrel, which would further hamper sediment penetration and restrict deployment of such a large device to muddy substrates.

The length of the core tube unit can also be adjusted according to the research needs and may range between 1 m and 3 m. The 1.5 m length we chose was a compromise between ease of handling (small boat) and the time span of several hundred to thousands of years that we hoped to cover. 16‐cm‐diameter cores longer than 3 m, although achievable, might substantially increase the infrastructure necessary for their handling; cores shorter than 1 m can also be gained using simpler equipment.

### Underwater monitoring system

Finally, a simple underwater monitoring system mounted on top of the tripod would help visualize the progress in sediment penetration (see also Blomqvist 1991). As shown in Fig. 4h, the corer has reached its final penetration depth when the upper end fitting of the slit‐rod is about 10 cm away from the slit‐head. In this position, the hydraulic core catcher is closed (or can be closed manually using the water hose), and extraction can proceed. Mounting a small cable‐connected camera capturing this detail would simplify the decision about the right moment to start retrieval.
